# On-Chip Optical Adder and Differential-Equation-Solver Based on Fourier Optics and Metasurface

**DOI:** 10.3390/nano12193438

**Published:** 2022-09-30

**Authors:** Yutai Chen, Huan Chen, Hansi Ma, Zhaojian Zhang, Wanlin Xie, Xin Li, Jian Chen, Junbo Yang

**Affiliations:** Center of Material Science, College of Sciences, National University of Defense Technology, Changsha 410073, China

**Keywords:** 4f system, analog optical computing, optical adder, optical differential equation solver, silicon-on-insulator, on-chip, direct-binary-search (DBS) algorithm

## Abstract

Analog optical computing (AOC) has attracted great attention over the past few years, because of its ultra-high speed (potential for real-time processing), ultra-low power consumption, and parallel processing capabilities. In this article, we design an adder and an ordinary differential equation solver (ODE) on chip by Fourier optics and metasurface techniques. The device uses the 4f system consisting of two metalenses on both sides and one middle metasurface (MMS) as the basic structure. The MMS that performs the computing is the core of the device and can be designed for different applications, i.e., the adder and ODE solver in this article. For the adder, through the comparison of the two input and output signals, the effect of the addition can be clearly displayed. For the ODE solver, as a proof-of-concept demonstration, a representative optical signal is well integrated into the desired output distribution. The simulation result fits well with the theoretical expectation, and the similarity coefficient is 98.28%. This solution has the potential to realize more complex and high-speed artificial intelligence computing. Meanwhile, based on the direct-binary-search (DBS) algorithm, we design a signal generator that can achieve power splitting with the phase difference of π between the two output waveguides. The signal generator with the insertion loss of −1.43 dB has an ultra-compact footprint of 3.6 μm× 3.6 μm. It can generate a kind of input signal for experimental verification to replace the hundreds of micrometers of signal generator composed of a multi-mode interference (MMI) combination used in the verification of this type of device in the past.

## 1. Introduction

Powerful computing power, efficient information processing systems, and rapidly growing big data require the deployment of new approaches using photons rather than electrons as the primary information carriers to alleviate the bandwidth and speed bottlenecks that have arisen in traditional electronics. Optics provide a unique and productive platform for parallel analog computing. Optical computing has obvious advantages, such as ultra-high speed, ultra-low power consumption, and parallel computing capability [1]. Analog optics dedicated to a single function are developing rapidly with the leap in artificial intelligence. Since Silva et al. proposed spatial analog optics, some spatial domain devices, such asthe spatial filter, convolver, differentiator, integrator, etc., have been proposed [2]. Unlike time-domain devices, the signals in these devices are distributed in space. By using spatial Fourier transform methods or Green’s function methods, one can implement the desired transfer function to perform operations. As a new type of structure that can adjust the light field freely, the metasurface seems to be the perfect candidate. At present, a variety of analog optical computing devices have been designed in the airspaces through the structure of metasurfaces, such as metasurfaces designed by Green’s function method [3,4], transflective metasurfaces [5,6,7,8,9], photonic crystal metasurfaces [10,11,12], and multi-layer film metasurfaces [13,14,15]. However, assembling these metasurface systems in space is a very difficult task. It is very attractive to integrate such an optical system on a chip.

Previously, Zi Wang et al. designed an on-chip differentiator using Fourier optics and the metasurface by etching air slits on a silicon-on-insulator (SOI) [16]. The device reduces the size and can be integrated on the chip, which is easier to assemble, and it realizes an ideal differentiation function for on-chip spatial signals. With the same method, Chao Chen et al. fabricated an integrator on chip using the 4f system [17]. The device was verified through three different input signals, and the final result was excellent. However, as far as we known, the on-chip optical adder and the ordinary differential equation (ODE) solver have not been studied yet, which are common AOC devices.

In this article, based on the Fourier optics design method, we design an adder model and an ODE solver model on the on-chip 4f system. Both of the devices have a compact footprint of 60 μm× 14 μm. The input signal is transverse electric (TE) mode light at 1550 nm. Two identical metalenses are utilized to perform the Fourier transforms in a 4f system, and the specially designed middle metasurface (MMS) is embedded in the middle to carry out the operation of adder or ODE solver on the Fourier plane. For adder, by comparing the two input and output signals, the adding effect can be clearly displayed. For the ODE solver, in a proof-of-concept demonstration, the output distribution fits well with the expected curve with the similarity coefficient of 98.28%. Meanwhile, in order to generate a kind of input signal for experimental verification, we design a power divider with a phase difference of π between two waveguides by using the direct-binary-search (DBS) inverse algorithm. We reach a size as small as 3.6 μm× 3.6 μm. As an input-signal generator, it can replace the previous hundreds of micrometers’ multi-mode interference (MMI) combination with the same function in the experimental verification of on-chip 4f system [16,17], thereby greatly reducing the size of the device.

## 2. Operation Principle and Implementation Method

**Figure 1:**
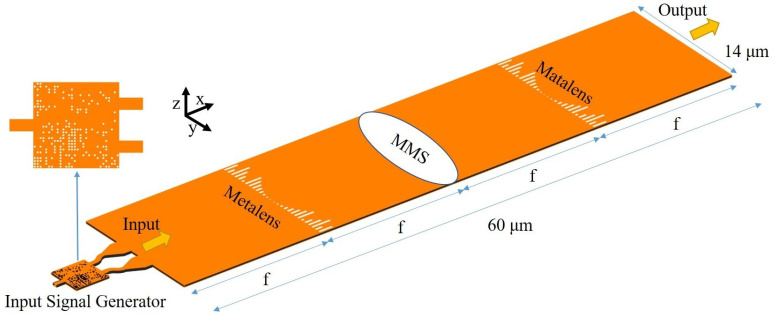
Schematic diagram of the overall structure of the device. The MMS is the core of the device and can be designed to implement various applications.

We use a spatial domain design method based on Fourier optics to design our structures. Figure 1 shows the schematic of our on-chip 4f system. ‘f’ is the focal length of the lens, and ‘4f’ means that the system has four focal lengths. The system consists of three metasurfaces, which are two identical metalenses and a MMS. Metasurfaces are artificial materials that can flexibly and effectively control the polarization, amplitude, phase, propagation mode, or other characteristics of electromagnetic waves. Its feature size is always smaller than the wavelength or the same order of magnitude as the wavelength. A metalens is a lens that use metasurfaces to focus light. In our 4f system, the first metalens performs Fourier transforms on the input signal produced by a signal generator, and the distribution of spatial frequency is presented on the middle Fourier plane. This spatial frequency is processed by the MMS and then converted back to the spatial domain by an inverse Fourier transform performed by the second metalens.(According to the theory of Fourier optics, the 4f system is designed in infinite free space, so this device is placed on a very wide slab waveguide. Figure 1 only shows its core part.) Therefore, we mainly elaborately control the structure of the MMS to make it satisfy the transmission function (amplitude of the transmittance and the phase shift distribution) of the calculation we desire and then finally realize the operation function.

The metalenses and the MMS have similar structures. They are composed of a series of air slits with different lengths and widths shown as the white rectangles in Figure 1. The air slit is the basic unit of the metasurfaces and is used to manipulate the transmission and phase-shift of the light by changing the length and width. The input light propagates along the x-axis. Different units with specific length/width shapes are arranged along the y-axis to construct the metasurface and is symmetric in the y-axis. The air slits are etched 220 nm in depth on the silicon layer. The center distance of adjacent units are 500 nm. By elaborately designing the width and length of each slit, the amplitude and phase shift of incident light can be properly manipulated and fulfill the requirements of the metalenses and MMS.

**Figure 2:**
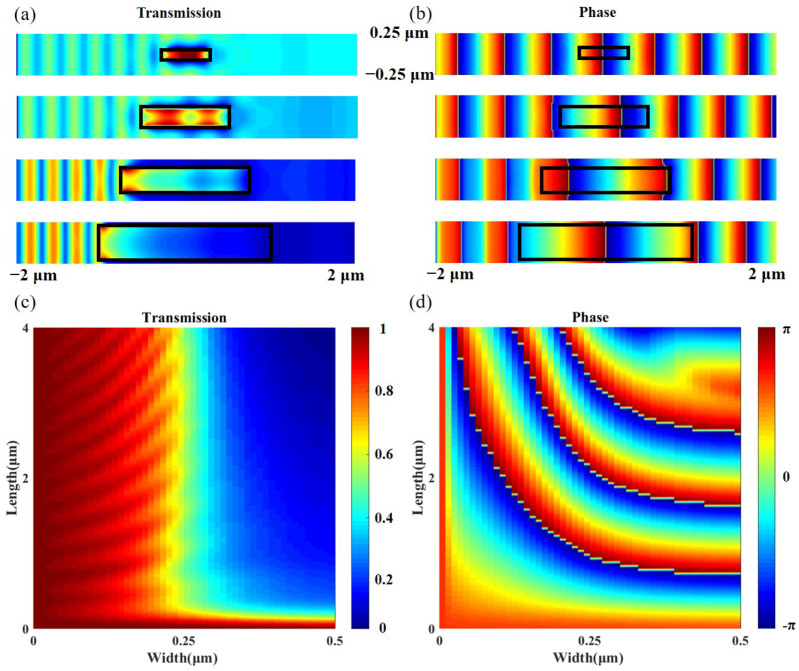
(**a**) Intensity distribution of $E_{y}$ near the air slit. The black rectangle is the outline of the slit. (**b**) Phase distribution near the air slit. (**c**) Simulated transmission modulation and (**d**) phase-shift modulation library for different slit widths and lengths. (To be noted, the phase here is continuously distributed. We change the phase and color at π and −π to better demarcate the 2π phase range and distinguish its phase interval).

We use the finite-difference time-domain (FDTD) method to simulate the symmetric slit structure. The refractive indices of Si and air at 1550 nm are 3.48 and 1, respectively. Figure 2a,b show the simulated transmission and phase under quasi-planar wave incidence at 1550 nm for different slits. The black rectanglular lines in Figure 2a,b show the borders of the air slits with different widths and lengths. From top to bottom, the widths and lengths are: 0.1/0.5, 0.2/1, 0.3/2, and 0.4/3 μm. We can see the electric field intensity and phase distributions are all flat, which indicates that the slit affects the output light field uniformly. Figure 2c,d show the transmission and phase shift modulation library for the slit with different widths from 0–0.5 μm and lengths from 0–4 μm. The transmission modulation range is 0.06–1, and the phase modulation range is up to 22 rad, which meets our design needs.

For metalenses, the transmission characteristic is only related to spatially phase shifts:

(1) $$\varphi \left(y\right)=\frac{2\pi }{\lambda }n_{\mathrm{eff}}\left(f-\sqrt{f^{2}+y^{2}}\right),$$

where $\lambda ,n_{\mathrm{eff}},f,and\;y$ represent the wavelength of the input signal, the effective refractive index, the focal length of the metalens, and the position on the lens in the y direction, respectively [18,19]. In order to meet the manufacturing requirements and maximize the transmission and phase-control capability, we set the width of each slit of the metalens to 0.14 μm, and the insertion loss of the metalens is kept below 0.84 dB. Based on Equation (1), the length of the slits at different positions is changed to match the phase-shift distribution required by Equation (1), then we obtain a metalens with a focal length of 15 μm in Figure 1. Next, based on the modulation diagrams we obtained, as well as the transmission and phase shift distributions of MMS required for transfer functions of different operations, we can design the adder and differential equation solver.

## 3. Input Signal Generator

To test an adder or ODE, an input signal generator is needed since the needed input signal is complex and not easy to be achieved. Here, we design a signal generator using the DBS algorithm [20,21,22,23]. The device can generate two beams of light with the same power and a phase difference of π. The size of the device is only 3.6 μm × 3.6 μm, which can replace the MMI combination of hundreds of microns used in the verification of such devices in the past [16,17].

**Figure 3:**
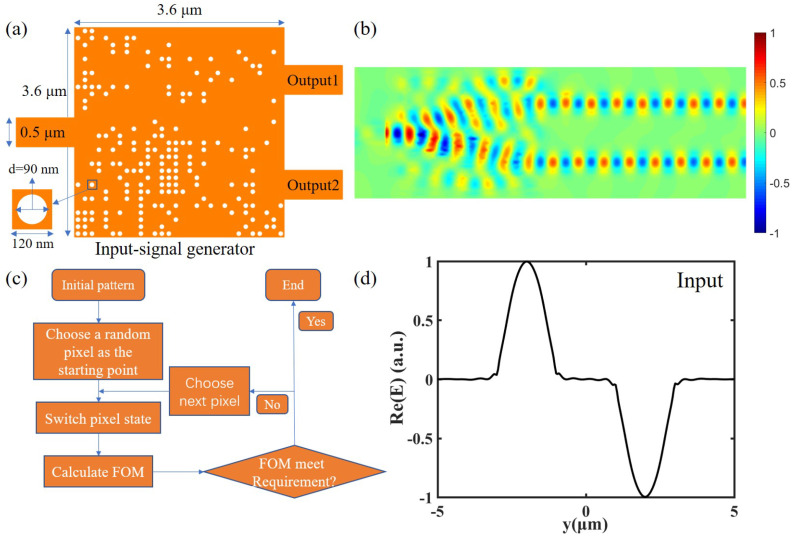
(**a**) Schematic diagram of the structure of the input-signal generator. (**b**) Simulated electric field distribution of the designed input-signal generator. (**c**) DBS algorithm flow chart. (**d**) Schematic diagram of the input signal generated by the device.

The DBS algorithm firstly discretizes the design area of the device into hundreds of basic pillars which are called pixels, and these pixels have two states, “etched” or “unetched”. The algorithm will randomly generate or manually set the initial structure of the pixel state distribution and then randomly select or specify the pixel to start the iteration and flip its state. The performance of the device is defined by a figure-of-merit (FOM) function. The higher the FOM, the better the performance. For each iteration, one pixel’s state is changed; by calculation of the FOM, the algorithm decides whether to keep the change of the pixel or return to its previous states. If the FOM goes higher, then it keeps, and, if not, it returns to its previous state. An iteration ends when all the pixels have been toggled and calculated. After many iterative calculations, the algorithm will continue calculating until the performance of the device is stable. The specific flow of the DBS algorithm is shown in Figure 3c.

For our input-signal generator, there are two output channels. The two beams of light in two channels have the same power and a phase difference of π. In order to realize the function, we set the FOM function of the device as:

(2) $$FOM=3\times \left(1-\left(\left(T_{1}-0.5\right)+2\times \left(T_{2}-0.5\right)\right)-\left|T_{1}-T_{2}\right|\right)-\left|P_{1}-P_{2}-\pi \right|,$$

where $T_{1}$ and $T_{2}$ represent the transmission of output1 and output2, $P_{1}$ and $P_{2}$ represent the phase of output1 and output2. Formula $\left(\left(T_{1}-0.5\right)+2\times \left(T_{2}-0.5\right)\right)$ means that the transmission of the two beams is larger and closer to 0.5. The coefficient ‘2’ is to increase the $T_{2}$ and decrease the influence of π phase shift for $T_{2}$. Formula $\left|T_{1}-T_{2}\right|$ means that the energy gap between the two beams is smaller. Formula $\left|P_{1}-P_{2}-\pi \right|$ means that the phase difference of the two beams should be closer to π. The coefficient ‘3’ at the start of the formula is to make the transmission part the same order of magnitude with the phase part.

By iterating the pixels, we obtain the optimized structure shown in Figure 3a. This device is designed on SOI substrates with a Si thickness of 220 nm and a ${\mathrm{SiO}}_{2}$ thickness of 1 μm. The input light of this device is TE polarized light at 1550 nm wavelength. It has an ultra-compact footprint of 3.6 μm× 3.6 μm and is divided into smaller square pixels with the size of 120 nm × 120 nm. Each pixel has two states, with or without a circular air hole in the center, shown as the white circular in Figure 3a. The diameters and depths of the circular air holes are 90 nm and 220 nm. The insert loss of this device is −1.43 dB. The transmissions of output1 and output2 are 0.361 and 0.358, respectively, which is approximately equal. The phase difference between two waveguides is 3.161 rad, which is basically the same as π. Figure 3b shows the electric field distribution of this device. We can clearly see that the wave is divided into two parts, and the phases are gradually staggered in the main part of device. Finally, the energies in the two waveguides are basically the same, and have a phase difference of π. The main purpose of this device is to replace the bulky signal generator used in the experimental verification of on-chip 4f system optical computing device in the past. Finally, this device can generate the input signal as shown in Figure 3d. We use the signal generated by this signal generator to simulate and obtain the corresponding results in the following adder and ODE solver. Similarly, by using the DBS algorithm, similar structures, and more ports and then calculating different FOM functions, we can design more signal generators that can generate various input signals.

## 4. Adder

For an ideal adder, there are two images on the input plane of the 4*f* system. Their centers are on the central axis of the lens, and the distances from the central axis are *a* and −*a*, respectively. When the two images pass through the adder, the effect of adding the two images can be seen in the center of the output plane. According to the addition property of the Fourier transform, the ideal transmission function is:

(3) $$T\left(y\right)=\frac{1}{2}\left(1+\cos\left(\frac{2\pi ay}{\lambda f}\right)\right),$$

where *a* is the distance from the central axis of the lens to the center of the waveguide of the two added signals, λ is the wavelength, and *f* is the focal length. We can verify this with the principles of Fourier optics. Assuming that there are two coherent plane waves of normalized amplitude incident on the input plane, the amplitude distribution of the two images on the first lens plane is:

(4) $$u\left(y_{1}\right)=u_{1}\left(y_{1}-a\right)+u_{2}\left(y_{1}+a\right).$$

Its frequency spectrum is U(ξ) on the MMS plane. The transmission function is T(ξ)=H(ξ), which can be regarded as the transfer function of the system, ξ=y/λf. So the amplitude of the output plane is:

(5) $$u\left(y_{3}\right)=F^{-1}\left(U\left(\xi \right)H\left(\xi \right)\right)=u\left(y_{3}\right)\times h\left(y_{3}\right),$$

$h\left(y_{3}\right)$ is the point spread function of H(ξ):

(6) $$h\left(y_{3}\right)=F\left(1+\cos\left(2\pi \xi \right)\right)=\delta \left(y_{3}\right)+\frac{1}{2}\delta \left(y_{3}-a\right)+\frac{1}{2}\delta \left(y_{3}+a\right).$$

So, the amplitude distribution of the output plane is:

(7) $$\begin{array}{cc} \; & u_{i}\left(y_{3}\right)=\left[u_{1}\left(y_{3}-a\right)+u_{2}\left(y_{3}+a\right)\right]\times \left[\delta \left(y_{3}\right)+\frac{1}{2}\delta \left(y_{3}-a\right)+\frac{1}{2}\delta \left(y_{3}+a\right)\right]\\ \; & =\frac{1}{2}\left[u_{1}\left(y_{3}\right)+u_{2}\left(y_{3}\right)\right]+\left[u_{1}\left(y_{3}-a\right)+u_{2}\left(y_{3}+a\right)\right]+\frac{1}{2}\left[u_{1}\left(y_{3}-2a\right)+u_{2}\left(y_{3}+2a\right)\right]. \end{array}$$

The first term of Formula (7) indicates that half of the sum of the two signals can be obtained at the middle of the output plane, and the other part indicates that there will be side lobes on both sides of the output plane center.

**Figure 4:**
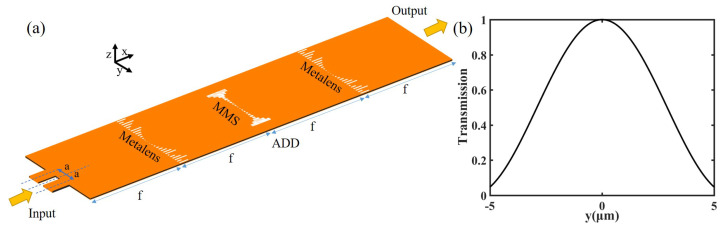
(**a**) Schematic diagram of the adder device structure. (**b**) The required transmission of the MMS in adder 4f system.

Next, we set *a* = 2 μm, λ = 1550 nm, *f* = 15 μm. The transmission function that the MMS needs to satisfy is shown in Figure 4b. We need to elaborately design the width and length of the slits in different period positions according to the transmission modulation library of Figure 2c, so that the MMS composed of different air slits can satisfy the above transmission function. Here, we use 20 air slits to implement the function curves. The corresponding lengths and widths of each air slit are shown in Table 1. Finally, our designed adder structure is shown in Figure 4a. The MMS structure shows a symmetrical distribution with a thin middle and thick sides, which can make the light passing through it have a higher transmission in the middle and a lower transmission on both sides, so as to conform to the transmission distribution in Figure 4b.

**Figure 5:**
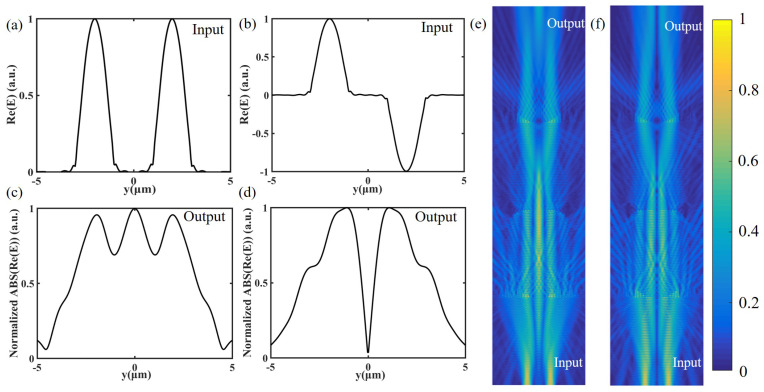
(**a**) Input signal when the two beams of light have no phase difference. (**b**) Input signal when the two beams of light have a phase difference of π. (**c**) Output signal corresponding to Input (**a**). (**d**) Output signal corresponding to Input (**b**). (**e**) Electric field distribution corresponding to Input (**a**) and output (**c**). (**f**) Electric field distribution corresponding to Input (**b**) and output (**d**).

We simulate the device with two different input signals as shown in Figure 5a,b. When two waveguides input two beams of light with no phase difference (Figure 5a), a peak can be seen in the middle of the output result (Figure 5c). There will be side lobes on both sides of the output center, and the peak intensity is basically half of the sum of the highest points of the side lobes, which is consistent with the theory. The effect of adding the two beams is shown in the electric field distribution (Figure 5e). There is a distinct peak in the center of the output. We can obtain the result of adding the two signals by placing a waveguide in the center of the output plane. When two beams of light have π phase difference (Figure 5b), the theoretical added output is 0. At this time, we can also see that it is basically 0 in the middle of the simulated output result (Figure 5d), and, in the corresponding electric field diagram (Figure 5f), the electric field in the middle of the output plane is nearly 0. The results perfectly fit the function of an adder.

## 5. ODE Solver

**Figure 6:**
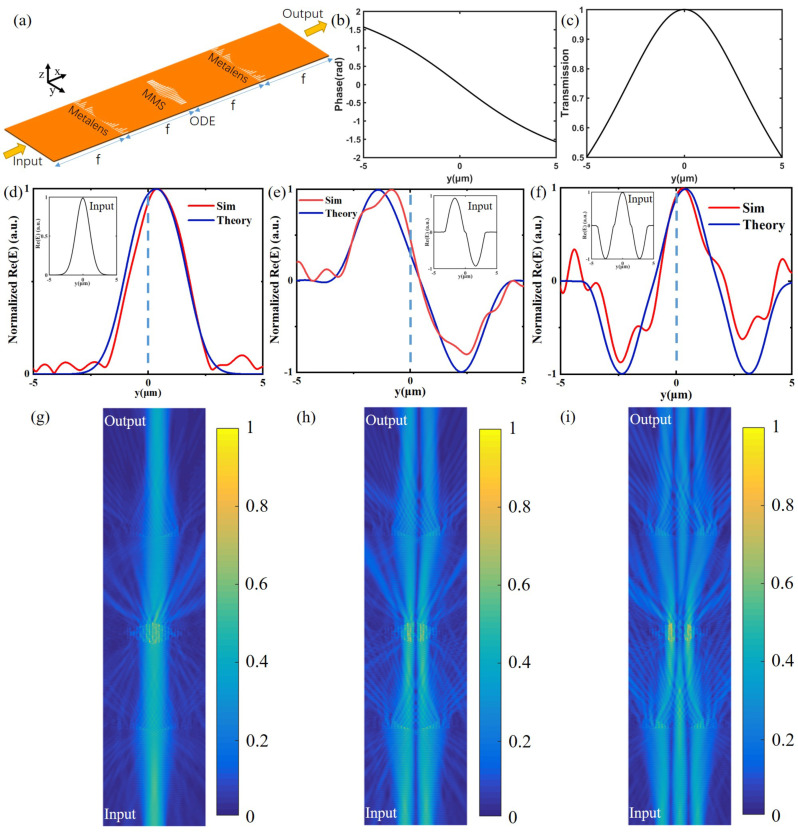
(**a**) Schematic diagram of the structure of the ode solver. (**b**) MMS phase-shift distribution. (**c**) MMS transmission distribution. (**d**–**f**) Normalized theory and simulation output of different input. The blue dotted line is the center of the picture, which can show the slight shift effect of the output result through comparison. Figure (**g**–**i**) are the schematic diagrams of the electric field corresponding to the Figure (**d**–**f**), respectively.

The OED solver is capable of modeling and controlling many fundamental physical phenomena and practical engineering systems. The common second-order constant coefficient ODE can be expressed as:

(8) $$\left(\alpha d^{2}g\left(y\right)/dy^{2}\right)+\left(\beta dg\left(y\right)/dy\right)+\gamma \left(g\left(y\right)\right)=f\left(y\right),$$

where f(y) denotes the integral input signal, g(y) denotes the solution of the equation (output signal), and (α,β,and γ) are positive constants of arbitrary values [1]. Using the Fourier transform, the transfer function in the spatial domain can be described as:

(9) $$T\left(y\right)=1/\left(\gamma +i\beta y-\alpha y^{2}\right),$$

where α,β,and γ are constants [1]. We multiply the numerator and denominator of this formula by $\left[\left(\gamma -\alpha y^{2}\right)-i\beta y\right]$ to separate the real and imaginary parts of the transfer function. The magnitude of this complex function is the transmission, and the argument is the phase shift. Although any arbitrary value can be applied to the constant coefficients, to simplify the transfer function, here we assume (α,β,γ) to be $\left(4/D^{2},4/D,1\right)$, D = 10 μm, respectively. After calculation, Figure 6b,c depict the transmission and phase-shift distribution of the transfer function above. Next, according to the transmission phase-shift modulation library (Figure 2c,d), we design the width and length of the slit at each period position of the MMS to meet the requirements of Figure 6b,c). The final structure is shown in Figure 6a. The MMS presents an approximately symmetrical distribution about the central axis, so as to realize the distribution of the symmetrical transmission (as shown in Figure 6c) around the central axis. The length in the central axis −*y* direction is overall slightly longer than the length in the *y* direction of the central axis, so that the phase shift distribution shown in Figure 6b is achieved. The length and width of each slit in the MMS are shown in Table 2.

To verify the performance of the device, we use a signal generator to input a signal and obtain a simulation output result. Through the Euler method of numerical analysis, we obtain the theoretical output signal. As is shown in Figure 6d, we compare the simulation output and theoretical output. The inset is the input signal. The signal is the electric field (Re(E)). The theoretical output waveform is shifted to the right slightly compared to the input waveform, and there is not much change overall (under the setting of our (α,β, and γ) coefficients, and, after many numerical analysis calculations and verifications, there are not many changes indeed). The simulated output waveform also exhibits a certain right shift and fits well with the theoretical output waveform. Figure 6g of the electric field distribution corresponding to this input signal also shows the effect that the output signal does not change much and shifts to the right slightly. We can roughly understand this phenomenon from another angle. As shown in Figure 6c, the transmission of MMS is symmetrical, which makes the overall change smaller. Figure 6b shows that the phase shift gradually decreases as *y* increases, that is, the phase shift in the −*y* direction is overall larger than the phase shift in the y direction and the largest difference is π. According to Fermat’s principle, the light will shift slightly to the right.

To evaluate the performance of this device, we define a similarity coefficient:

(10) $$R^{2}=1-SS_{\mathrm{res}\;}^{2}/SS_{\mathrm{tot}\;}^{2},$$

where $SS_{\mathrm{res}}^{2}$ and $SS_{\mathrm{tot}}^{2}$ denote residual sum of squares and total sum of squares, respectively [17]. After calculation, the similarity coefficient of the simulation output and theoretical output is 98.28%.

To further investigate the feasibility of this ODE solver, more simulations were performed using different input signals. As shown in Figure 6e,f, by adjusting the input signal, we can compare the theoretical output and simulated output. The simulated output curves fit well with the theoretical output curve, and both do not change much and shift slightly to the right compared to the input. Figure 6h,i are the electric field diagrams corresponding to Figure 6e,f, respectively. The electric field at the output is basically the same as the electric field at the input and slightly shifts to the right in two diagrams, which is consistent with the output waveform. Finally, the similarity coefficients between the simulated and theoretical results are 94.86% and 84.95% for Figure 6h,i, respectively.

It should be noted that there is a certain deviation between the theoretical output and the simulation output. This is because the boundary condition on both sides of the slit is the periodic boundary condition when we measure the transmission and phase shift in the single-slit simulation. However, in order to meet the transmission and phase-shift requirements of different positions of the MMS, the sizes of adjacent slits are different. The periodic boundary condition for the simulation of a single slit is no longer satisfied, which results in the transmission and phase shift of each slit in MMS being slightly different from those of the same size slit in the modulation table of Figure 2c,d. Finally, there is a difference between the designed transmission function $T{\left(y\right)}^{\prime }$ and the ideal transmission function T(y). There are two ways to solve this problem. The first is to reduce the size difference between adjacent slits and try to choose slits in the same phase interval, which is the divided block in the Figure 2d. In fact, the phase here is continuously distributed, just to make it all between −π and π, so it is divided into multiple blocks. We try to keep each slit in the same phase interval, so that the size difference between adjacent slits can be reduced to obtain something closer to a periodic boundary condition. The second is to use the DBS algorithm, genetic algorithm, particle swarm algorithm, adjoint method, and other algorithms to design MMS. Aiming at the ideal transmission and phase-shift distribution, we can strengthen the integrity of the MMS, reduce the isolation of the MMS composed of discrete slit, and finally achieve a better effect.

Whether it is an adder, an ODE solver, or a previously designed differentiator and integrator, these devices can only perform single-function calculations. In order to achieve more calculations on a same structure, we can turn air slits into phase-change material slits, and then place one or more electrodes next to each small slit to control the refractive index of the phase-change material. In this way, we can change and control the MMS transmission phase-shift distribution to meet the MMS transmission functions required by different calculations. Then, based on the ultrafastness, parallel processing, and low-loss properties, the ideal multi-function high-speed optical computing effect can be realized, which paves the way for future big data processing and artificial intelligence ultra-high-speed computing.

## 6. Discussion

Due to the fact that the designed devices are models temporarily, it is necessary to analyze certain fabrication deviations. At present, electron beam lithography (EBL) can realize 1 nm precision lithography. Additionally, micro/nano fabrication technology has reached a very high level. Here, we assume that the diameter of the holes and the length and width of the slits will have a fabrication deviation of +6 nm and −6 nm and analyze the impact on the device performance.

**Figure 7:**
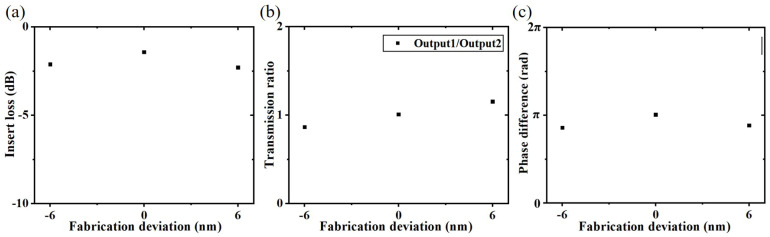
When the hole-diameter fabrication deviation is 0 nm, −6 nm, or 6 nm, (**a**) the insertion loss of the device, (**b**) the transmission ratio of Output 1 and Output 2, and (**c**) the phase difference of two channels.

For the signal generator, we simulate the devices with diameter deviations of +6 nm and −6 nm, respectively and obtain the results as shown in Figure 7. Figure 7a shows that when the fabrication deviation is −6 nm and +6 nm, the device insert loss slightly decreases from −1.43 dB to −2.12 dB and −2.29 dB, respectively. Figure 7b shows that the ratios of Output1 and Output2 transmission change from 1.01 to 0.87 and 1.15, respectively, when the fabrication deviation is −6 nm and +6 nm, which indicates that the energy of the two ports is still basically equal, and the transmission ratios will fluctuate slightly around 1. As shown in Figure 7c, the phase differences are 2.7 rad at −6 nm and 2.78 rad at +6 nm, which shows that the phase difference will change slightly around π. The above analysis indicates that the performance change of the signal generator is acceptable at least within the range of 6 nm hole-diameter fabrication deviation.

Meanwhile, many devices with similar structures and different functions have been designed and fabricated. Through experimental verification, their output results are consistent with the simulation, and the device performance is excellent [24,25,26,27]. There is no problem in the fabrication of the devices with such structures.

**Figure 8:**
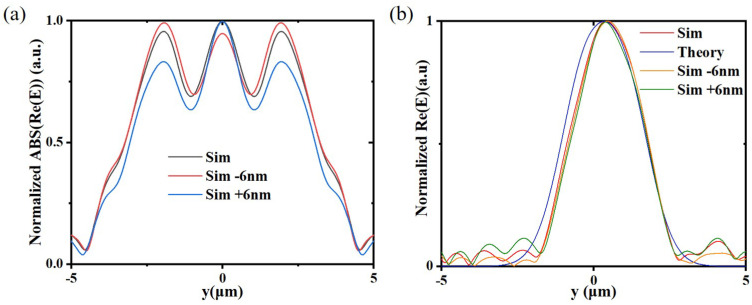
When the lengths and widths of all slits are widened or shortened by 6 nm, (**a**) the adder output result with the input signal of Figure 5a, and (**b**) the ODE solver output result with the input signal of the insert of Figure 6d.

For the adder and OED solver, we select an input signal, respectively, to analyze the impact of fabrication deviation on performance. As previously stated, we assume that the lengths and widths of all slits are widened or shortened by 6nm. After simulation, we obtain the normalized results as shown in Figure 8. For the adder, we select the input signal of Figure 5a. As shown in Figure 8a, when the fabrication deviations are −6 nm and +6 nm, compared to the side lobe, the middle peak slightly decreases and rises, respectively, but the effect of adding is obvious. After that, the side lobes will be filtered out, and we will finally obtain the output result through a waveguide located in the middle of the output plane, so the fabrication deviation within at least 6 nm has little impact on the device. For the ODE solver, we select the input signal of the insert of Figure 6d. As shown in Figure 8b, the output results are only slightly different when the fabrication deviations are −6 nm and +6 nm, which indicates that this device has good robustness within the range of 6 nm fabrication deviation.

Similarly, same types of devices have been verified in experiments, such as on-chip differentiators [16] and on-chip integrators [17]. Their output results are consistent with the simulation and have good effects. The feature sizes of our devices are basically the same as theirs, so our device fabrication and experiment can also be successfully completed.

## 7. Comparison

As shown in Table 3, in the previous on-chip 4f system experimental verification for the differentiator and integrator, to generate a signal that can output two beams of light with the same power and π phase difference, an MMI combination with a footprint of tens of microns × hundreds of microns was used. In this article, through etching 90 nm cylindrical air holes on SOI, we use the DBS algorithm to design a signal generator with a ultra-compact footprint of only 3.6 μm× 3.6 μm that can generate the same input signal with transmissions of 0.361 and 0.358 for output 1 and output 2, respectively.

As shown in Table 4, compared with the previous on-chip differentiator and on-chip integrator, we realize new functions of the adder and ODE solver on chip, and the footprint is approximately the same. At the same time, for testing, we design a new signal generator with the DBS algorithm. The device is only 3.6 $\mu m^{2}$, which is far smaller than the previously designed MMI combined signal generator with hundreds of square microns, and can generate the same signal.

## 8. Conclusions

We designed an on-chip optical adder model and an ODE solver model based on the SOI platform. We used a symmetry-slit structure to form three metasurfaces in a 4f system. The footprint of the two devices is 60 μm× 14 μm with the focal length of 15 μm. Through verification, the simulation output is in good agreement with the theoretical output under different input light field signals. The adder and ODE solver can perform parallel computing and processing with ultra-high speed and ultra-low power consumption. Meanwhile, through the DBS algorithm, we design an input-signal generator to generate two beams of light with the same power and a phase difference of π. The generator is only 3.6 μm× 3.6 μm, which can replace the MMI combination of hundreds of microns used in the verification of such devices in the past. Using the adder and the ODE solver design method, we can design more devices, such as subtractors and integral-differential equation (IDE) solvers. This solution is promising to realize more complex and ultra-high speed artificial intelligence computing.

## Figures and Tables

**Table 1:** Length (L (μm)) and width (W (μm)) parameters of each slit (from −y to *y*) in the adder MMS.

L:	3	1.9	0.9	0.35	0.45	0.3	0.25	0.2	0.1	0.1
W:	0.49	0.41	0.35	0.35	0.26	0.24	0.21	0.16	0.29	0.07
L:	0.1	0.1	0.2	0.25	0.3	0.45	0.35	0.9	1.9	0.3
W:	0.07	0.29	0.16	0.21	0.24	0.26	0.35	0.35	0.41	0.49

**Table 2:** Length (L (μm)) and width (W (μm)) parameters of each slit (from −y to *y*) in the adder MMS.

L:	1.8	1.84	1.88	1.92	2.075	2.36	2.45	3	2.96	3.525
W:	0.25	0.24	0.23	0.22	0.2	0.17	0.16	0.12	0.12	0.09
L:	3.215	2.93	2.7	2.1	1.85	1.65	1.45	1.39	1.32	1.25
W:	0.1	0.11	0.12	0.16	0.18	0.2	0.22	0.23	0.24	0.25

**Table 3:** Comparison of input signal generators in different articles.

Reference	Design Method	Material	Size	1:1 Beam-Splitting Phase Difference
[17]	MMI combination	SOI (Si: 220 nm)	Tens of microns × hundreds of microns	— π
[16]	MMI combination	SOI (Si: 220 nm)	Tens of microns × hundreds of microns	— π
This article	DBS algorithm	SOI (Si: 220 nm)	3.6 μm × 3.6 μm	Output1: 0.36 Output2: 0.358 π

**Table 4:** Comparison of same-type devices in different articles.

Reference	Material	Footprint	Function	Signal Generator
[16]	SOI(on-chip)	45 μm × 20 μm	Differentiator	MMI combination of hundreds of square microns
[17]	SOI(on-chip)	67 μm × 14 μm	Integrator	MMI combination of hundreds of square microns
This article	SOI(on-chip)	60 μm × 14 μm	Adder, ODE solver	Metamaterial structure of 3.6 $\mu m^{2}$ by DBS algorithm

## Data Availability

Not applicable.
